# Gadolinium-enhanced MRI reveals dynamic development of endolymphatic hydrops in Ménière's disease^☆^

**DOI:** 10.1016/j.bjorl.2018.10.014

**Published:** 2018-12-20

**Authors:** Xuanyi Li, Qianru Wu, Yan Sha, Chunfu Dai, Ru Zhang

**Affiliations:** a National Health Commission Key Laboratory of Hearing Medicine (Fudan University), Department of Otology and Skull Base Surgery, Shanghai, China; b Hospital of Xuzhou Medical University, Department of Otolaryngology, Xuzhou, China; c Eye, Ear, Nose and Throat Hospital, Fudan University Department of Radiology Shanghai, Shanghai, China; d Shanghai East Hospital, Department of Otorhinolaryngology Shanghai, Shanghai, China

**Keywords:** Endolymphatic hydrops, Meniere disease, Magnetic resonance imaging, Gadolinium, Injection, Intratympanic, Hidropsia endolinfática, Doença de Ménière, Imagem de ressonância magnética, Gadolínio, Injeção, Intratimpânica

## Abstract

**Introduction:**

Meniere's disease is associated with impaired hearing, tinnitus, vertigo, and aural fullness. Many anatomical studies have suggested idiopathic endolymphatic hydrops as the pathological basis of Meniere's disease, which now can be visualized by using gadolinium -enhanced magnetic resonance imaging of the inner ear.

**Objective:**

To investigate the development of endolymphatic hydrops in Meniere's disease by monitoring the vestibules and cochleae of affected patients.

**Methods:**

Inner ears of 178 patients with definite unilateral Meniere's disease diagnosis were visualized by 3-dimensional fluid-attenuated inversion recovery and three-dimensional real inversion recovery magnetic resonance imaging following bilateral gadolinium intratympanic injection. The scans were used to evaluate the presence and degree of endolymphatic hydrops in the vestibules and cochlear structures, including the cochlear apical turn, the cochlear middle turn, and the cochlear basal turn. The correlation of endolymphatic hydrops occurrence between the various parts of the inner ear was determined.

**Results:**

Symptomatic endolymphatic hydrops was detected on the affected side in all patients, whereas asymptomatic endolymphatic hydrops was detected on the unaffected contra-lateral side in 32 patients (18.0%). On the affected side, the cochlear apical turn and the cochlear middle turn demonstrated significantly higher rates of endolymphatic hydrops than the cochlear basal turn and the vestibule. The severity of endolymphatic hydrops gradually decreased from the cochlear apical turn to the cochlear basal turn. On the contra lateral side, the incidence and degree of the detected asymptomatic endolymphatic hydrops were significantly greater in the cochleae than in the vestibules (*p *< 0.05), with no significant difference detected between the cochlear turns.

**Conclusion:**

Progression of endolymphatic hydrops appears to be directional, initiated in the cochlea. The order of endolymphatic hydrops severity gradually decreases from the cochlear apical turn to the cochlear basal turn and then to the vestibule. Endolymphatic hydrops in the vestibule is associated with symptomatic Meniere's disease.

## Resumo

**Introdução:**

A doença de Ménière está associada a deficiência auditiva, zumbido, vertigem e plenitude auricular. Muitos estudos anatômicos sugerem hidropsia endolinfática idiopática como a base patológica da doença, que agora pode ser visualizada através de estudo por imagem da orelha interna por ressonância magnética com gadolínio.

**Objetivo:**

Investigar o desenvolvimento da hidropsia endolinfática na doença de Ménière com monitoramento dos vestíbulos e das cócleas dos pacientes afetados.

**Métodos:**

Orelhas internas de 178 pacientes com diagnóstico definitivo de doença de Ménière unilateral foram visualizados através de imagem de recuperação de inversão atenuada por fluidos em ressonância magnética tridimensional, 3-D FLAIR, e por inversão real após injeção intratimpânica bilateral de gadolínio. Os exames foram usados para avaliar a presença e o grau de hidropsia endolinfática nos vestíbulos e nas estruturas cocleares, inclusive o giro coclear apical, o giro coclear médio e o giro coclear basal. A correlação da ocorrência de hidropsia endolinfática entre as várias partes da orelha interna foi determinada.

**Resultados:**

Hidropsia endolinfática sintomática foi detectada no lado afetado em todos os pacientes, enquanto hidropsia endolinfática assintomática foi detectada no lado contralateral não afetado em 32 pacientes (18,0%). No lado afetado, o giro apical da cóclea e o giro coclear médio demonstraram taxas significativamente mais altas de hidropsia endolinfática do que o giro basal e o vestíbulo. A gravidade da hidropsia endolinfática diminuiu gradualmente do giro apical da cóclea para o giro basal. No lado contralateral, a incidência e o grau da hidropsia endolinfática assintomática detectada foram significantemente maiores nas cócleas do que nos vestíbulos (*p* < 0,05), sem diferença significante entre os giros cocleares.

**Conclusões:**

A progressão da hidropsia endolinfática parece ser direcional, iniciando-se na cóclea. A sua ordem da gravidade diminui gradualmente do giro apical da cóclea para o giro basal e, em seguida, para o vestíbulo. A hidropsia endolinfática no vestíbulo está associada à doença de Ménière sintomática.

## Introduction

Meniere's disease (MD), first reported in 1861 by Prosper Ménière, is associated with impaired hearing, tinnitus, vertigo, and aural fullness.^1^ The syndrome is caused by disturbances in the homeostasis of the inner ear, resulting in impaired cochlear and vestibular functions. Since the first reports of endolymphatic hydrops (EH) in the temporal bones of patients with MD, many anatomical studies, lacking identification of causal abnormalities, have suggested idiopathic EH as the pathological basis of MD.^2^, ^3^, ^4^, ^5^ Various factors, including viral infections,^6^, ^7^ autoimmune disorders^8^, ^9^ and endocrine malfunctions,^10^ have been associated with EH development. However, some studies have suggested that EH is not a direct etiological factor in the development of MD and the emergence of MD symptoms.^11^, ^12^ Investigations of the role of EH in MD are still hampered by a limited understanding of EH development *in vivo*.

The current gold standard for EH detection is autopsy analysis of temporal bones, which generally detects developed EH and fails to analyze the progression of EH. Nakashima et al.^13^ first visualized the endolymphatic space in living patients using Gadolinium (Gd)-enhanced 3-Dimensional Fluid-Attenuated Inversion Recovery (3D-FLAIR) magnetic resonance imaging (MRI) of the inner ear. Since then, 3D-FLAIR MRI and 3-Dimensional real Inversion Recovery (3D-real IR) MRI have been commonly used for EH analysis.^14^, ^15^ Human and animal studies revealed that 3D-real IR imaging provides more clear and consistent histopathological observations than 3D-FLAIR imaging.^15^, ^16^ However, 3D-FLAIR imaging outperforms 3D-real IR under conditions of insufficient Gd delivery into the perilymphatic space following an intratympanic injection.^16^

This study utilized Gd-enhanced inner ear MRI to investigate the development of EH in patients with MD. Specifically, 3D-FLAIR and 3D-real IR MRI scans were used to analyze the occurrence and degree of EH in the vestibule and each cochlear turn of the inner ears of MD patients.

## Patients and methods

### Patients

This study evaluated 178 patients (110 males and 68 females) with definite unilateral MD diagnosis according to the 1995 American Academy of Otolaryngology-Head and Neck Surgery guidelines.^17^ Median patient age was 52 years (23–74 years), and median MD duration was 3.7 years (2 months, 20 years). The enrollment criteria for the present study were: unilateral disease, two or more definitive episodes of vertigo with hearing loss, tinnitus, or aural fullness and no history of middle ear or neurological disorders. All individuals complained of hearing loss on the affected side. All patients were diagnosed by the corresponding author in the Department of Otology and Skull Base Surgery of the Eye, Ear, Nose and Throat Hospital at Fudan University between July 2013 and May 2017. MD stage was determined based on the worst audiogram in the 6 months prior to MRI scanning, using the pure-tone average calculated at the thresholds of 0.5, 1, 2, and 3 kHz.^17^

The study was approved by the medical ethics committee of the Eye, Ear, Nose and Throat Hospital at Fudan University (2014007), and all patients signed informed consent forms.

### MRI

The patients received bilateral intratympanic injections of Gd diluted in saline (v/v 1:7) using a 22 gauge spinal needle and a 1 mL syringe. Gadopentetate dimeglumine was chosen in this research as the Gd contrast agent. Following the injections, the patients maintained an upright seated position for 30 min without speaking or swallowing. After 24 h MRI scans were performed using a 3 T MR unit (Verio, Siemens Healthcare, Erlangen, Germany) with a 32 channel phased-array receive-only head coil.

T2-space, 3D-real IR, and 3D-FLAIR sequence MRI images were collected as previously described.^14^ Briefly, the parameters for the 3D-real IR sequence were as follows: voxel size of 0.4 × 0.4 × 0.8 mm, scan time of 14 min, repetition time (TR) of 9000 ms, echo time (TE) of 181 ms, inversion time (TI) of 1730 ms, slice thickness of 0.80 mm, field of view (FOV) of 160 × 160 mm, and matrix size of 3300 × 918. The parameters for 3D-FLAIR sequence were as follows: voxel size of 0.7 × 0.7 × 0.6 mm, scan time of 6 min, TR of 6000 ms, TE of 387 ms, TI of 2100 ms, slice thickness of 0.60 mm, echo train length of 173, FOV of 220 × 220 mm, and matrix size of 1701 × 810.

### Image evaluation

After intratympanic injection, Gd permeates into the perilymph through the oval and round windows to the inner ear. Therefore, perilymphatic space with accumulated Gd is detectable as a contrast-enhanced signal, whereas endolymphatic space demonstrates a negative signal. Analysis of the boundary between the perilymphatic and the endolymphatic spaces facilitates detection of EH, evidenced as an enlarged negative signal expanding into the positive signal of the perilymphatic space confined to the bony labyrinth.

An experienced radiologist, with no information about the clinical diagnosis, assessed the MRI scans for EH degree as none, mild, or significant according to the three-stage grading criteria^18^ (Table 1). Adobe Photoshop CS5 (Adobe Systems, Inc., San Jose, CA) was utilized to calculate the ratio of the endolymphatic space to the vestibular fluid space.

**Table 1:** Three-stage grading of endolymphatic hydrops using magnetic resonance imaging.

Hydrops stage	Vestibule^a^	Cochlea
None	Area ratio ≤ 1/3	No displacement of Reissner's membrane.
Mild	1/3 < Area ratio < 1/2	Displacement of Reissner's membrane, with endolymphatic space area not exceeding scala vestibule area.
Significant	Area ratio > 1/2	Endolymphatic space area exceeds scala vestibule area.

a The ratio of the area of the endolymphatic space to the area of the vestibular fluid space (sum of the areas of the endolymphatic and the perilymphatic spaces).

### Statistical analysis

Statistical analysis was performed using SPSS 20.0 (IBM, Armonk, NY). McNemar's test, Wilcoxon matched-pairs signed-ranks test, and Spearman's rank correlation test were used to analyze the data. The level of significance was set at *p* < 0.05.

## Results

### Detection of EH

**Figure 1:**
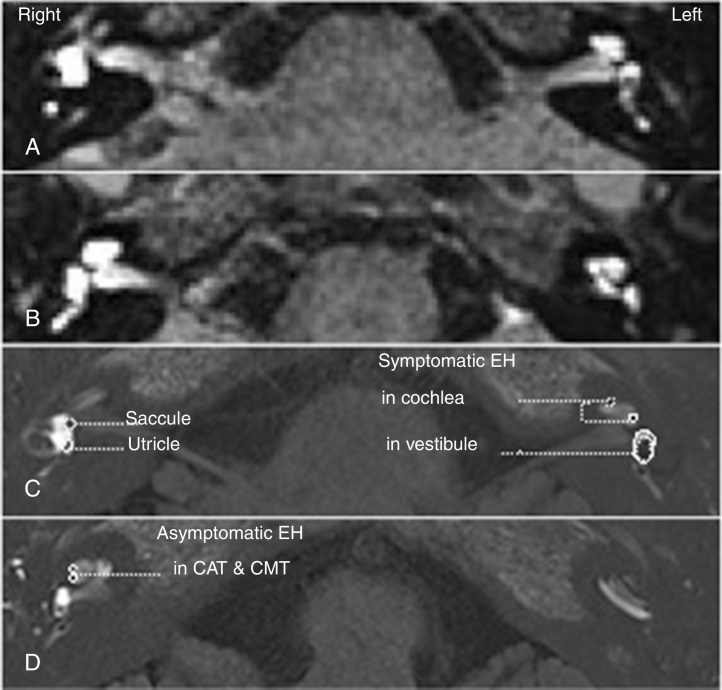
Bilateral endolymphatic hydrops (EH) was detected in a patient diagnosed with left unilateral Meniere's disease (MD). Three-dimensional fluid-attenuated inversion recovery (3D-FLAIR) (A, B) and three-dimensional real inversion recovery (3D-real IR) (C, D) magnetic resonance imaging (MRI) was performed 24 hours following a bilateral intratympanic gadolinium (Gd) injection. 3D-FLAIR MRI showed bilateral perilymphatic space permeated with Gd (A, B). EH was present in bilateral cochleae (C, D) and the left vestibule (A, C), but absent in the right vestibule (B, C, D). Symptomatic EH was present in all turns of left cochlea, while asymptomatic EH was only present in apical and middle turn (CAT & CMT) (C, D). The degree of symptomatic EH in the left cochlea (affected side) was greater than that of the asymptomatic EH in the right cochlea (unaffected side) (C, D). Significant EH was observed in the left vestibule, in which the boundary between the saccule and the utricle could not be detectable (C). The saccule and utricle could be detected respectively in the right vestibule, in which no EH was present (C).

Cochleae and vestibules were detectable on MRI scans obtained 24 h following bilateral intratympanic injections of the contrast agent. 3D-FLAIR and 3D-real IR sequences were used for the detection and evaluation of EH in the vestibules and cochlear structures, including the Cochlear Apical Turn (CAT), the Cochlear Middle Turn (CMT), and the Cochlear Basal Turn (CBT) of the MD patients (Fig. 1). Among the 178 patients diagnosed with unilateral MD, asymptomatic EH was detected in the contralateral unaffected inner ear of 32 patients (18.0%)

EH commonly occurred in all analyzed anatomical structures in the affected inner ears (Table 2). Specifically, EH was detected in CATs, CMTs, CBTs, and vestibules on the affected side in 97.2%, 97.2%, 94.9%, and 95.5% of the 178 patients, respectively. Among the 32 unilateral patients of MD with bilateral EH, in contrast, the asymptomatic EH on the asymptmatic side was detected in 96.9%, 96.9%, 90.6%, and 31.3% of CATs, CMTs, CBTs and vestibules, respectively (Table 3).

**Table 2:** Endolymphatic hydrops (EH) in various parts of the symptomatic inner ears of 178 patients with unilateral Ménière's disease.

	Vestibule+	Vestibule−	Total	CAT+	CAT−	Total
CAT+	165 (92.7%)	8 (4.5%)	173 (97.2%)			
CAT−	5 (2.8%)	0 (0.0%)	5 (2.8%)			
Total	170 (95.5%)	8 (4.5%)	178 (100%)^a^			
CMT+	165 (92.7%)	8 (4.5%)	173 (97.2%)	173 (97.2%)	0 (0.0%)	173 (97.2%)
CMT−	5 (2.8%)	0 (0.0%)	5 (2.8%)	0 (0.0%)	5 (2.8%)	5 (2.8%)
Total	170 (95.5%)	8 (4.5%)	178 (100%)^a^	173 (97.2%)	5 (2.8%)	178 (100%)^c^
CBT+	162 (91.0%)	7 (3.9%)	169 (94.9%)	168 (94.4%)	1 (0.6%)	169 (94.9%)
CBT−	8 (4.5%)	1 (0.6%)	9 (5.1%)	5 (2.8%)	4 (2.2%)	9 (5.1%)
Total	170 (95.5%)	8 (4.5%)	178 (100%)^b^	173 (97.2%)	5 (2.8%)	178 (100%)^a^

CAT, Cochlear apical turn; CMT, Cochlear middle turn; CBT, Cochlear basal turn; +, represents detection of EH in indicated structure; −, represents absence of EH in indicated structure.

a CAT vs. Vest, CMT vs. Vest, CBT vs. CAT, *p* = 0.000.

b CBT vs. Vest, *p* = 0.188.

c CMT vs. CAT, *p* = 1.000.

**Table 3:** Endolymphatic hydrops (EH) in various parts of the contra-lateral asymptomatic ears of 32 patients with bilateral Ménière's disease.

	Vestibule+	Vestibule−	Total	CAT+	CAT−	Total
CAT+	9 (28.1%)	22 (68.8%)	31 (96.9%)			
CAT−	1 (3.1%)	0 (0.0%)	1 (3.1%)			
Total	10 (31.3%)	22 (68.8%)	32 (100%)^a^			
CMT+	9 (28.1%)	22 (68.8%)	31 (96.9%)	31 (96.9%)	0 (0.0%)	31 (96.9%)
CMT−	1 (3.1%)	0 (0.0%)	1 (3.1%)	0 (0.0%)	1 (3.1%)	1 (3.1%)
Total	10 (31.3%)	22 (68.8%)	32 (100%)^a^	31 (96.9%)	1 (3.1%)	32 (100%)^c^
CBT+	7 (21.9%)	22 (68.8%)	29 (90.6%)	29 (90.6%)	0 (0.0%)	29 (90.6%)
CBT−	3 (9.4%)	0 (0.0%)	3 (9.4%)	2 (6.3%)	1 (3.1%)	3 (9.4%)
Total	10 (31.3%)	22 (68.8%)	32 (100%)^a^	31 (96.9%)	1 (3.1%)	32 (100%)^b^

CAT, Cochlear apical turn; CMT, Cochlear middle turn; CBT, Cochlear basal turn; +, represents detection of EH in indicated structure; −, represents absence of EH in indicated structure.

a CAT vs. Vest, CMT vs. Vest, CBT vs. Vest, *p* = 0.000.

b CBT vs. CAT, *p* = 0.125.

c CMT vs. CAT, *p* = 1.000.

On both sides, the prevalence of EH in the CAT was similar with that in the CMT (*p* = 1.000). On the affected side, EH occurred more frequently in the CAT and the CMT than in the vestibule or the CBT (McNemar's test, *p* < 0.05). There was no statistical significance in the rates of EH detection between the vestibules and the CBTs (Table 2).

On the contra-lateral unaffected side, asymptomatic EH was significantly less common in the vestibule than in the cochlea (Table 3) (vestibule vs. CAT, vestibule vs. CMT, vestibule vs. CBT, *p* = 0.000). No significant differences in EH rates were detected between the cochlear turns. Moreover, vestibular EH was detected at significantly higher rates on the affected side (95.5%) than on the contralateral side (31.3%).

### Degree of EH

To examine the relationship between disease progression and degree of EH (Table 1), we classified the MD stage of each patient according to pure tone threshold calculations. Thus, eight patients were classified as Stage 1, 20 patients as Stage 2, 109 patients as Stage 3, and 41 patients as Stage 4. Considering the small number of individuals with early stage MD, we combined Stages 1 and 2 for the analysis. In addition, we performed separate analyses for the symptomatic and the contralateral asymptomatic sides of the 32 patients with bilateral MD.

**Figure 2:**
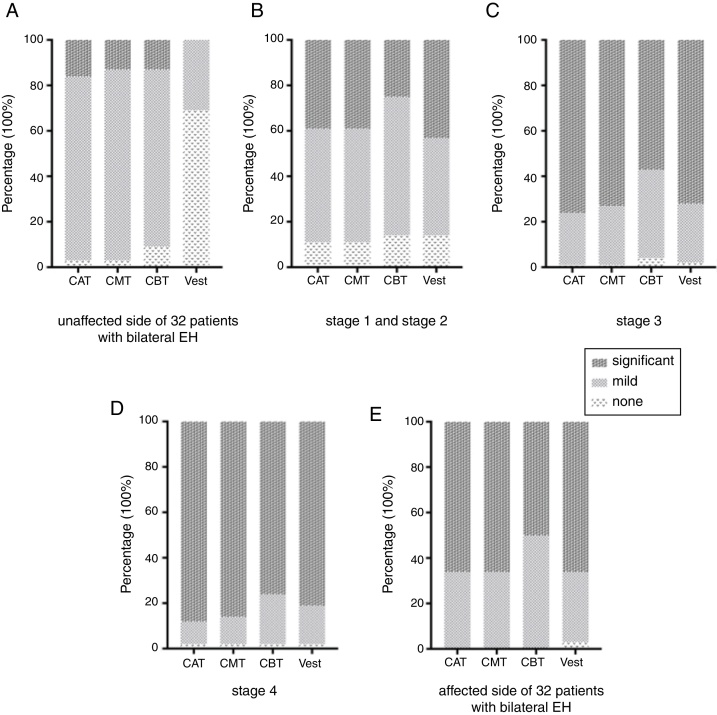
Percent distribution of endolymphatic hydrops (EH) degrees in different groups classified according to Meniere's disease (MD) stages and lateral symptoms. (A) Asymptomatic EH on contra-lateral unaffected side of 32 patients with bilateral EH. (B) EH on the affected side of 28 patients with Stage 1 and Stage 2 MD. (C) EH on the affected side of 109 patients with Stage 3 MD. (D) EH on the affected side of 41 patients with Stage 4 MD. (E) EH on the affected side of 32 patients with bilateral EH. EH was classified as undetectable (none), mild, or significant. Data are presented as percentages of EH detected at a certain degree. CAT, Cochlear apical turn; CMT, Cochlear middle turn; CBT, Cochlear basal turn; Vest, Vestibule.

In patients with bilateral EH, the cochleae of the contralateral asymptomatic sides demonstrated the presence of predominantly mild EH, with very low rates of significant EH detected (Fig. 2A). The vestibules of the asymptomatic sides were predominantly free of detectable EH, with no significant EH observed. Thus, although no differences in EH degree were detected between the cochlear turns, the EH degree profiles of the vestibules and the cochlea were significantly different (Table 4).

**Table 4:** Comparison of endolymphatic hydrops degree at different Ménière's disease stages (*p*-value).

Anatomical structure	Asymptomatic contra-lateral EH	Stage 1 + 2	Stage 3	Stage 4
	*n* = 32	*n* = 28	*n* = 109	*n* = 41
CAT vs. CMT	0.317	1.000	0.083	0.317
CAT vs. CBT	0.180	0.059	0.000^a^	0.059
CAT vs Vest	0.000^a^	0.978	0.234	0.317
CMT vs. CBT	0.157	0.059	0.000^a^	0.102
CMT vs. Vest	0.000^a^	0.978	0.499	0.527
CBT vs. Vest	0.000^a^	0.197	0.009^a^	0.593

CAT, Cochlear apical turn; CMT, Cochlear middle turn; CBT, Cochlear basal turn; Vest, Vestibule.

a *p* < 0.05.

In contrast to the asymptomatic contralateral sides, the symptomatic sides of early stage MD demonstrated substantial rates of both mild and significant EH in the vestibule (Fig. 2B). In addition, symptomatic ears also demonstrated less differences in EH degree profiles of the cochlear turns and the vestibules than did the asymptomatic ears (Fig. 2A–D) (Table 4). The progression of MD from Stage 1 to Stage 4 corresponded with increasing severity of EH in each part of the inner ear (Fig. 2B–D). For all classified MD stages, significant EH was the least common in the CBT relative to the other cochlear turns (Fig. 2A–D). However, patients with Stage 4 MD exhibited significant EH in all parts of the inner ear, with no significant differences detected among any two parts (Fig. 2D) (Table 4). In addition, we confirmed that the degree of symptomatic EH on the affected side of the 32 patients with bilateral EH was not overall milder and was similar to the symptomatic EH on the affected side of all 178 patients.

### The correlation of EH occurrence

On the symptomatic side of the patients diagnosed with unilateral MD, a significant correlation of EH occurrence was observed between any two parts of the inner ear. The correlation between the different cochlear turns was higher than that between the vestibule and the cochlea. The highest coefficient was observed for the comparison between the CAT and the CMT. On the contra-lateral asymptomatic side of the 32 patients with bilateral EH detected in this study, the correlation of asymptomatic EH among the cochlear turns was consistent with that observed for the affected side. Specifically, the correlation between the CAT and the CMT was greater than that between the CMT and the CBT (Spearman's rank correlation test, *p* < 0.05). On the contrary, no significant correlation between the vestibule and any of the cochlear turns was detected on the unaffected side (Table 5).

**Table 5:** Correlation between endolymphatic hydrops (EH) occurrence in the vestibules and cochlear turns.

	Symptomatic side of MD patients (*n* = 178)				Contra-lateral asymptomatic side of patients with bilateral EH (*n* = 32)			
	CAT	CMT	CBT	Vest	CAT	CMT	CBT	Vest
CAT	1	0.949^a^	0.674^a^	0.447^a^	1	0.903^a^	0.628^a^	0.234
CMT		1	0.686^a^	0.424^a^		1	0.863^a^	0.155
CBT			1	0.442^a^			1	−0.065
Vest				1				1

MD, Ménière's disease; CAT, Cochlear apical turn; CMT, Cochlear middle turn; CBT, Cochlear basal turn; Vest, Vestibule.

a *p* < 0.01

## Discussion

MD is associated with recurrent episodic vertigo, tinnitus, aural fullness, and fluctuating hearing loss.^19^ It is a multifactorial disorder of the inner ear characterized by endolymphatic hydrops, or a distended endolymphatic space, which is detectable in temporal bones upon autopsy analysis. Animal models have been used to explore the mechanisms underlying MD and EH.^20^, ^21^ However, evidence regarding the role of EH as a causative factor of MD or an epiphenomenon during MD progression has remained inconclusive.^22^ To our knowledge, the *in vivo* development of EH in MD patients remains poorly understood. Temporal bone autopsy, considered a gold standard for the detection of EH, cannot be used to monitor the pathogenetic process or to control for such variables as disease onset.

By enabling the visualization of the boundary between the endolymphatic and perilymphatic spaces, MRI scanning following intratympanic or intravenous Gd injection facilitates the detection of EH in living patients.^23^, ^24^, ^25^ In this study, we performed Gd-enhanced MRI to visualize occurrence and degree of EH in different parts of inner ear, and also related the stages of patients with the severity of EH to analyze and deduce the dynamic development of EH in population.

All 178 patients with definite unilateral MD tested in this study had EH in the vestibule or the cochlea of the affected symptomatic ear. Asymptomatic EH was also detected in the contralateral unaffected ears of 32 patients. Asymptomatic EH appeared more frequently in the cochleae than in the vestibules. Previous studies utilizing temporal bone autopsy or temporal bone Gd-enhanced MRI have similarly reported asymptomatic EH. Following temporal bone analysis in children, Bachor and Karmody^26^ reported low rates of saccule and utricle dilation but high incidence of bulging in the cochlear duct, with 65.6% occurring in the apical turn. Recent studies have also reported asymptomatic EH in the vestibules and cochleae of unaffected ears, though at rates different from those observed in our study.^25^, ^27^ These studies have suggested that bilateral EH does not necessarily correlate with clinical signs of MD on both sides. Evidence of asymptomatic EH has been used to support the view that EH is a feature, rather than a cause, of MD.^11^, ^12^ However, the detection of EH via temporal bone autopsy in 100% of patients with a history of MD supports the hypothesis that EH causes MD.^22^

Thus, we sought to investigate the relationship between EH and the symptoms of MD. Our results emphasized the differences between EH in the symptomatic ear and the contralateral asymptomatic ear. Although cochlear EH was prevalent on both sides, differences in EH rates between the cochlea and the vestibule were more substantial on the asymptomatic side than on the affected side. Asymptomatic EH was detected in 31.3% of the vestibules on the unaffected side, whereas 95.5% of the symptomatic side vestibules exhibited signs of EH. The degree of EH was also significantly different between the two sides. The degree of asymptomatic EH on the contra-ateral side was much milder than that of the symptomatic EH on the affected side. In particular, the difference in EH degree in the vestibules of asymptomatic ears and those of symptomatic early-stage MD ears underscored the role of vestibular EH in the clinical symptoms of MD. Based on the analysis above, presence of EH in any part of inner ear, especially in cochlear turns, was not necessary for onset of MD symptoms, so EH should be identified as the pathological basis of MD, instead of the causal and definitive factor. However, as the severity of EH in vestibule progressively worsened, potentially in conjunction with other factors, the onset of MD symptoms was more likely to occur.^21^, ^28^ This result was much consistent with Yoshida T et al.,^29^ in 2018. Yoshida T reported that EH was present in the cochlea of 45/52 affected ears of patients with MD (87%) and in 16/42 control ears which were not associated with MD (38%). However, in the vestibule, symptomatic EH was present in 49/52 affected ears (94%) and asymptomatic EH in 3/42 control ears (7%). Consequently, Yoshida T et al., regarded EH in the vestibule as a more specific predictor of definite MD than EH in the cochlea. Similarly, the results in this manuscript would also emphasize the importance of EH in vestibule to the onset of MD symptoms, especially the severity of EH in vestibule.

As for the susceptibility of EH, our observations revealed that, on both sides, EH occurred more commonly and at a higher degree of severity in the cochlea, especially in the apical turn, than in the vestibule. This result is consistent with the temporal bone autopsy findings of Okuno and Sando, demonstrating that EH is more commonly observed in the pars inferior (cochlea and saccule) than in the pars superior (utricle and semicircular canals) of the temporal bone.^30^ As to the higher susceptibility of EH in cochlea, we could refer it to the distribution of dark cells in the inner ear. Kimura RS^31^ reported that dark cells in the vestibule are mainly localized in the ampullae, utricle and common crus, which was not detected in the saccule, saccular duct, ductus reuniens and main parts of the canals by the combined use of macroscopic observations, light microscopy and electron microscopy. Due to the characteristics of the distribution of vestibular dark cells, and the morphology and function of Bast's valve, the endolymph in he saccule should be generated most likely from the dark cells of the stria vascularis in cochlea. Thus, we emphasized the importance of the stria vascularis in cochlea in the pathogenesis of MD, which may produce redundant endolymph.

Multiple studies have provided evidence for the susceptibility of the apical turn to significant EH.^32^, ^33^ Nageris et al. reported that varying degrees of displacement of the basilar membrane towards the scala tympani in the apical turn, which was not observed in other turns of the cochlea, may be related to atrophy of the spiral ligament.^32^ Valk et al. reported that, in the guinea pig, a rupture of Reissner's membrane in response to destructive acute endolymphatic hydrops most often occurs in the apical turn of the cochlea.^33^ As for the higher susceptibility of significant EH in apex in the cochlea, we are not sure that it is the cause of hearing loss in early MD, which typically starts with low-frequency tones, even though low-frequency tones are consistent with the location of the basilar membrane in apex turn.^19^

Based on the observed relationships between MD stage and EH distribution and degree, we propose that EH undergoes dynamic development in patients with MD. Considering that the incidence of asymptomatic EH was much lower in the vestibule than in the cochlea and was very similar between the cochlear turns, we propose that EH initially develops in the cochlea. During the asymptomatic stage, EH is likely mild in both the cochlea and the vestibule. With disease progression, the severity of EH gradually increases, especially in the vestibule, and the clinical symptoms of MD become apparent. Considering the prevalence of EH in the apical and middle turns of the cochlea and the high EH correlation observed for every two adjacent anatomical parts, we propose that EH originates in the CAT, with EH severity decreasing from the apical to the basal turn, and then from the cochlea to the vestibule.

Limitations should be noted in the interpretation of this study. Although this study demonstrated the occurrence and the distinct features of asymptomatic EH on the unaffected side of some MD patients, it has not assessed the incidence of asymptomatic EH in the general population and the potential progression of asymptomatic EH into MD.

## Conclusion

Dynamic development of EH appears to be directional, likely being initiated in the cochlea. The degree of EH severity gradually decreases from the CAT to the vestibule, with significant EH more likely to appear in the CAT and the CMT. Moreover, occurrence of EH in the vestibule is significantly correlated with symptomatic MD.

## Funding

10.13039/501100001809National Natural Science Foundation of China, 81570917, 81600802, 81771009.

## Conflicts of interest

The authors declare no conflicts of interest.
